# Co-dominant neutralizing epitopes make anti-measles immunity resistant to viral evolution

**DOI:** 10.1016/j.xcrm.2021.100257

**Published:** 2021-04-20

**Authors:** Allison J. Greaney, Frances C. Welsh, Jesse D. Bloom

**Affiliations:** 1 Basic Sciences Division and Computational Biology Program, Fred Hutchinson Cancer Research Center, Seattle, WA 98109, USA; 2 Department of Genome Sciences & Medical Scientist Training Program, University of Washington, Seattle, WA 98195, USA; 3 Molecular and Cellular Biology Graduate Program, University of Washington and Fred Hutchinson Cancer Research Center, United States, Seattle, WA 98195, USA; 4 Howard Hughes Medical Institute, Chevy Chase, MD 20815, USA

## Abstract

Munoz-Alia and colleagues^1^ demonstrate that neutralizing antibody immunity to measles resists viral evolutionary escape because it targets numerous distinct viral epitopes. Their work contributes to our understanding of what determines whether a virus can evolve to evade immunity.

## Main text

An enduring mystery is why some viruses undergo rapid antigenic evolution while others are more antigenically stable. The answer is more complicated than viral mutation rate. Influenza and measles viruses are both RNA viruses with similarly high mutation rates.^2^ However, influenza evolves antigenically to erode antibody immunity, whereas measles is antigenically stable such that a vaccine developed over a half-century ago still provides full protection against all currently circulating measles strains.

One hypothesis is that the surface proteins of some viruses are more functionally tolerant of mutations than others. Indeed, the surface proteins of rapidly evolving viruses such as influenza hemagglutinin are quite tolerant of mutations,^3^ whereas the measles virus hemagglutinin (H) protein is less mutationally tolerant.^4^ However, this hypothesis does not seem to be the whole story, since it is possible to select measles virus mutants that escape neutralization by individual monoclonal antibodies.^5^

**Figure 1:**
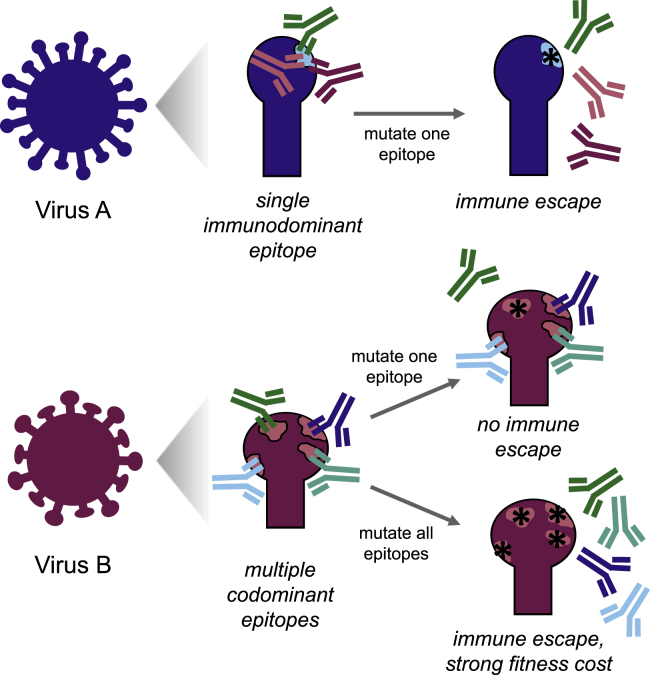
The polyclonal antibody response to a virus can be focused or broad In this example, virus A has a single immunodominant epitope, such that a single mutation greatly reduces antibody neutralization. In contrast, the multiple codominant epitopes in virus B make immune escape highly unlikely, as this would require a large number of mutations that may have fitness costs.

In a new study, Muñoz-Alía and colleagues^1^ demonstrate that characteristics of the polyclonal antibody response to measles virus play an important role in constraining viral evolution. The neutralizing activity of the polyclonal antibody response to infection or vaccination can be either narrowly focused on one or a few immunodominant epitopes, or broadly reactive to multiple codominant epitopes (Figure 1). For influenza virus, the polyclonal neutralizing antibody response is narrowly focused, such that single viral mutations can reduce neutralization by 10-fold or more.^6^ Muñoz-Alía and colleagues show that in contrast, the neutralizing antibody response to measles virus targets numerous codominant epitopes.

Specifically, Muñoz-Alía and colleagues^1^ employ an elegant series of mutagenesis experiments to demonstrate that the ability of the measles virus H glycoprotein to escape neutralizing antibodies is constrained by multiple codominant epitopes. They use *in vitro* escape selections to identify measles virus variants with mutations that escape neutralization by monoclonal antibodies targeting each of the eight distinct epitopes on the H protein. They then introduce mutations to each of these epitopes one-by-one and in combination and test how they affect neutralization by polyclonal serum antibodies. Their results show that ablation of at least five codominant epitopes is required to observe a substantial decrease in neutralization by polyclonal serum directed to the H protein. Furthermore, they demonstrate that the H protein itself is codominant for viral neutralization with the other major surface glycoprotein (F), such that mutations to both proteins are required for large drops in viral neutralization.

Thus, the existence of numerous codominant neutralizing epitopes constrains the antigenic evolution of measles virus. While a virus like influenza can often gain a large immune escape benefit via just a single mutation,^6^ measles virus may require five or more specific mutations to gain a comparable benefit. Even for a mutation-prone RNA virus, acquiring five specific mutations is an extraordinarily low probability event—especially because, as Muñoz-Alía and colleagues^1^ report, these combinations of escape mutations are highly functionally deleterious.

These results have important implications as we think about the potential for antigenic evolution of new viruses, such as SARS-CoV-2. Early in the pandemic, some suggested that coronaviruses were likely to be antigenically stable (like measles virus) because they have a lower mutation rate than other RNA viruses due to possessing a polymerase with “proofreading” activity.^2^ But the result of Muñoz-Alía and colleagues^1^ shows that mutation rate is just one factor affecting viral antigenic evolution, and the potential for antigenic evolution also depends on the immunodominance of the polyclonal neutralizing antibody response. Unfortunately, this response to coronaviruses appears to be narrowly focused such that single viral mutations can have large effects on polyclonal antibody neutralization^7^^,^^8^ in a manner more similar to influenza than measles virus. This narrow focusing of the neutralizing antibody response is probably a major factor enabling the antigenic evolution of SARS-CoV-2^9^ and other human coronaviruses.^10^

More generally, the work of Muñoz-Alía and colleagues^1^ suggests that to counter viral evolution, we should strive to develop vaccines that elicit antibodies targeting multiple distinct neutralizing epitopes and thus may be resistant to escape through viral antigenic evolution, like natural anti-measles immunity. Of course, this is easier said than done! But progress could come from building on the comparative studies described above to develop an improved mechanistic understanding of why the neutralizing antibody response to some viruses targets numerous codominant epitopes, whereas for others it is highly focused on just a single immunodominant epitope.
